# Progress and Challenges in Microalgal Biodiesel Production

**DOI:** 10.3389/fmicb.2016.01019

**Published:** 2016-06-30

**Authors:** Nirupama Mallick, Sourav K. Bagchi, Shankha Koley, Akhilesh K. Singh

**Affiliations:** ^1^Agricultural and Food Engineering Department, Indian Institute of Technology Kharagpur, KharagpurIndia; ^2^Amity Institute of Biotechnology, Amity University at Lucknow, LucknowIndia

**Keywords:** biodiesel, drying, harvesting, lipid yield, mass cultivation, microalgae, strain selection, transesterification

## Abstract

The last decade has witnessed a tremendous impetus on biofuel research due to the irreversible diminution of fossil fuel reserves for enormous demands of transportation *vis-a-vis* escalating emissions of green house gasses (GHGs) into the atmosphere. With an imperative need of CO_2_ reduction and considering the declining status of crude oil, governments in various countries have not only diverted substantial funds for biofuel projects but also have introduced incentives to vendors that produce biofuels. Currently, biodiesel production from microalgal biomass has drawn an immense importance with the potential to exclude high-quality agricultural land use and food safe-keeping issues. Moreover, microalgae can grow in seawater or wastewater and microalgal oil can exceed 50–60% (dry cell weight) as compared with some best agricultural oil crops of only 5–10% oil content. Globally, microalgae are the highest biomass producers and neutral lipid accumulators contending any other terrestrial oil crops. However, there remain many hurdles in each and every step, starting from strain selection and lipid accumulation/yield, algae mass cultivation followed by the downstream processes such as harvesting, drying, oil extraction, and biodiesel conversion (transesterification), and overall, the cost of production. Isolation and screening of oleaginous microalgae is one pivotal important upstream factor which should be addressed according to the need of freshwater or marine algae with a consideration that wild-type indigenous isolate can be the best suited for the laboratory to large scale exploitation. Nowadays, a large number of literature on microalgal biodiesel production are available, but none of those illustrate a detailed step-wise description with the pros and cons of the upstream and downstream processes of biodiesel production from microalgae. Specifically, harvesting and drying constitute more than 50% of the total production costs; however, there are quite a less number of detailed study reports available. In this review, a pragmatic and critical analysis was tried to put forward with the on-going researches on isolation and screening of oleaginous microalgae, microalgal large scale cultivation, biomass harvesting, drying, lipid extraction and finally biodiesel production.

## Introduction

‘Algae-for-fuel’ has gained renewed interest during the last decade. Unlike terrestrial feed-stocks such as soybean, rapeseed, jatropha, etc., microalgae have been projected with various advantages as (i) algae cultivation does not need arable land, (ii) higher photosynthetic rate of microalgae than terrestrial plants, (iii) microalgal oil yield could significantly exceed the yield of the best oilseed crops, (iv) microalgae can grow in seawater, brackish water or even wastewater, (v) microalgae cultivation can be combined with waste CO_2_ sequestration, (vi) use of the spent biomass for other value-added products, etc.

In these days, governments in various countries including the US, Italy, China, Germany, and India have been funding biofuel projects. For instance, the US alone has announced a spur package of US$100 billion to develop clean, alternative, and renewable technologies. Subsidies and incentives are also been introduced for vendors producing biofuels. Emissions must fall substantially and rapidly if we are to limit global climate change to below two degrees Celsius. Various reports emphasize that algae-derived biofuel can reduce CO_2_ emissions by fifty per cent as compared to the petroleum-based fuels. The ability of biofuels produced from algae to reduce environmental pollution significantly is, in turn, driving the research for the development of algae-biofuel technologies. However, there remain many challenges which are to be addressed to make algae-biofuel a commercial reality. In this review, we will address those challenges briefly.

## Microalgal Lipid Accumulation/Yield

At the outset, there is a need for high lipid-rich strains. Microalgae usually produce different ratios of lipids, carbohydrates, and proteins, which varies from species to species. However, physiological stresses such as nutrient limitations/deficiencies, salt stress, and high light intensity have been applied for directing metabolic fluxes to lipid biosynthesis of microalgae, thus, high lipid productivity can be achieved.

Nitrogen limitation/deficiency has been found to be the most suitable stimulant to raise lipid content of a number of microalgal species profoundly. Cellular lipid content reaching above 50% of dry cell weight (dcw) has been seen in a number of microalgal species (**Table 1**). In *Choricystis minor*, lipid content of 60% (dcw) against 27% control was recorded under simultaneous nitrate and phosphate deficiencies (Sobczuk and Chisti, 2010). Our laboratory batch culture study with *Scenedesmus obliquus* demonstrated lipid accumulation of 58% (dcw) under simultaneous nitrate and phosphate limitations in the presence of sodium thiosulphate against 13% control (Mandal and Mallick, 2009). With *Chlorella vulgaris*, simultaneous nitrate, phosphate, and iron limitations stimulate the lipid accumulation up to 57% against 8% control (Mallick et al., 2012). Li et al. (2013) observed an accumulation up to 60% in *Parachlorella kessleri* under nutrient limitations. Gorain et al. (2013) also reported lipid recovery of ~40% (dcw) in *Chlorella vulgaris* and *Scenedesmus obliquus* under osmotic stress. In *Nannochloropsis* sp., lipid content up to 60% (dcw) was observed with 15% CO_2_ sparging under nitrogen starvation and high light intensity (Jiang et al., 2011). The high light intensity with nitrogen- depleted conditions also found to raise the lipid content up to 54% (dcw) in *Nannochloropsis oceanica* IMET1 (Xiao et al., 2015). Recently, *Chlorella vulgaris* showed a lipid content of 63% when grown in municipal wastewater under nitrogen-limitation approach (Robles-Heredia et al., 2015). 61% lipid content in *Nannochloropsis gaditana* 1049 was observed by Hu et al. (2015), grown in seawater medium with Vitamin B12, thiamine, biotin supplementation and 5% CO_2_ sparging.

**Table 1:** Reports showing lipid content ≥50% (dry cell weight) in microalgae under specific culture conditions.

Microalga	Culture condition	Lipid content (% dcw)	Reference
*Dunaliella* sp.	1 M NaCl	71	Takagi et al., 2006
*Scenedesmus obliquus*	Nitrogen and phosphorus limitations in presence of thiosulphate	58	Mandal and Mallick, 2009
*Nannochloropsis oculata* NCTU-3	2% CO_2_ sparging	50	Chiu et al., 2009
*Neochloris oleoabundans*	Nitrogen limitation	56	Gouveia and Oliveira, 2009
*Nannochloropsis* sp. F&M-M24	Nitrogen deficiency	60	Rodolfi et al., 2009
	Phosphorus deficiency	50	
*Choricystis minor*	Nitrate and phosphate limitations	60	Sobczuk and Chisti, 2010
*Chlorella protothecoides*	Nitrogen deficiency under heterotrophy with sweet sorghum hydrolysate	50	Gao et al., 2010
*Chlorella zofingiensis*	Nitrogen limitation	55	Feng P. et al., 2011
*Isochrysis zhangjiangensis*	Elevated nitrogen addition (0.9%)	53	Feng D. et al., 2011
*Nannochloropsis* sp.	15% CO_2_ sparging under nitrogen starvation and high light intensity	60	Jiang et al., 2011
*Chlorella vulgaris*	Nitrogen starvation with 2–3% CO_2_ sparging	53	Mujtaba et al., 2012
*Nannochloropsis* sp.	32 g L^-1^ salinity with continuous CO_2_ sparging	52	Moazami et al., 2012
*Chlorella vulgaris*	Nitrogen, iron, and phosphorus limitations	57	Mallick et al., 2012
*Scenedesmus* sp. strain R-16	Nitrogen deficiency	53	Ren et al., 2013
*Tetraselmis* sp. MUR-233	Semi-continuous cultivation with CO_2_ sparging	55	Raes et al., 2014
*Chlorella pyrenoidosa*	Nitrogen deficiency	50	Fan et al., 2014
*Nannochloropsis oceanica* IMET1	High light intensity and nitrogen depleted culture conditions	54	Xiao et al., 2015
*Nannochloropsis salina*	Limited nitrogen addition	56	Fakhry and Maghraby, 2015
*Chlorella vulgaris*	Municipal wastewater utilization with nitrogen-limitation approach	63	Robles-Heredia et al., 2015
*Nannochloropsis gaditana* 1049	Seawater medium with Vitamin B12, thiamine, biotin, and air with 5% CO_2_ sparging	61	Hu et al., 2015

Physiological stresses or nutrient deficiencies/limitations are thus, employed to stimulate lipid accumulation in microalgae. However, under such conditions, they fail to grow well. The biomass productivity under such conditions is generally too low for the practical production of biodiesel from the cell mass. A general countermeasure to overcome this difficulty is to use a two-stage cultivation strategy, dedicating the first stage for cell growth/division in nutrient-sufficient medium, and the second stage for lipid accumulation under nutrient starvation or other physiological stresses. However, the major bottleneck here is a lack of a cost-effective harvesting technology for separation of the biomass two times, i. e., after the cultivation phase (first phase) to transfer the organisms to the lipid accumulation phase, and also after the lipid accumulation phase (second phase) for further processing. To overcome this, one strategy could be supplementation of the medium with dividing doses of major nutrients such as nitrate and phosphate, so to maintain the biomass yield and create a partial deficient/starved condition to stimulate the cellular lipid accumulation. Our laboratory study on this strategy gave full success for *Scenedesmus obliquus* and partial for *Chlorella vulgaris* and *C. minutissima*. Thus, further refinement in experimentation for *Chlorella* species is going on in this line to maximize the lipid yield under one-stage cultivation strategy. Therefore, the major challenges here are to find the ways/means to achieve high lipid productivity under one-stage cultivation, which would ultimately have a significant impact on the overall production cost of the algal fuel.

## Metabolic Engineering Approach to Increase Lipid Accumulation in Microalgae

In the era of advanced biodiesel production from microalgae, it is now indispensable to focus on the explorations of metabolic engineering using the techniques of molecular biology and genetic engineering. Metabolic engineering is commonly used for the purposeful alteration or introduction of a new gene or transcript factor to any targeted metabolic biochemical pathways for a particular organism by using recombinant DNA technology (Stephanopoulos, 1999). Implementations of the genetic engineering techniques in microalgal biodiesel production have gained a great interest, resulting in improved lipid yield, particularly the triacylglyceride (TAG) biosynthesis in microalgae. Microalgae metabolic engineering figured the origin for the fourth generation biodiesel production with an aim of transgenic microalgae development (Lu et al., 2011). Understanding of microalgal lipid biosynthetic pathway is of immense significance for the optimal and desired production of biodiesel. It should be well understood how the lipid metabolism was regulated by the enzymes and genes before any transgenic approach for any microalgal strain. Until today, lipid metabolism with a detailed description of biosynthetic pathways that modify the chain length or saturation/unsaturation of fatty acids has not been meticulously examined for microalgae as investigated for many other terrestrial plants. However, scientists reported that many genes responsible for the lipid metabolism in higher plants have also found to be homologous with the microalgal genome sequences (Radakovits et al., 2010).

In microalgal lipid metabolism, the free fatty acids are synthesized in the chloroplast, whereas the TAG biosynthesis is taken place in the endoplasmic reticulum. These two distinct pathways assembled and connected in the cytosol to form the TAG-lipid body. In the very first step of the fatty acid biosynthesis pathway, the acetyl-coenzyme A (acetyl-CoA) is converted into malonyl-CoA by the enzyme acetyl-CoA carboxylase (ACCase). Although many attempts have been made to overexpress the action of ACCase to induce the rate of lipid metabolism, a complete success story in this approach is yet awaited. Dunahay et al. (1996) was successful to overexpress the native ACCase (two to threefold rise) in diatom *Cyclotella cryptic* but no rise in lipid yield was seen in the transgenic diatom. In *Chlamydomonas reinhardtii*, the endogenous gene source of the fatty acid-ACP thioesterase (FATE) enzyme was modified for the nuclear overexpression; shorter chain length fatty acids were found with the endogenous gene as compared to the wild-type *C. reinhardtii* (Blatti et al., 2012). In a recent study, the antisense c-DNA modifications were achieved in the protein of pyruvate dehydrogenase kinase (PDH) for the diatom *Phaeodactylum tricornutum*. The PDH protein is responsible for the production of acetyl-CoA from pyruvate. In this approach, an overall 82% increase in neutral lipid content as compared to the wild-type strains was achieved (Ma et al., 2014). Zhang et al. (2014) in the same year targeted one lipogenesis transcription factor responsible for the free fatty acid biosynthesis in microalga *Chlorella ellipsoidea*. The nuclear overexpression was achieved with the source transcription factor obtained from soybean seed and the total lipids were increased by 52% against its wild type. Xue et al. (2015) studied the overexpression of mailc enzyme in *Phaeodactylum tricornutum*. The total lipid content in the transgenic *P. tricornutum* was increased significantly, and reached 57.8% (dcw) without hampering the growth rate. **Table 2A** provides an overview of these approaches.

**Table 2A:** Metabolic engineering approaches for enhanced fatty acid biosynthesis in microalgae.

Target enzyme/protein	Gene source/gene modification technique	Receiver species	Findings	Reference
Acetyl-CoA carboxylase (ACCase)	Endogenous	*Cyclotella cryptic*	No increase in overall lipid accumulation	Dunahay et al., 1996
Fatty acid-ACP thioesterase (FATE)	Endogenous	*Chlamydomonas reinhardtii*	Shorter chain length free fatty acids	Blatti et al., 2012
Pyruvate dehydrogenase kinase (PDH-K)	Antisense c-DNA modifications	*Phaeodactylum tricornutum*	Eighty-two percentage increase in neutral lipid content	Ma et al., 2014
One lipogenesis transcription factor	Soybean seed	*Chlorella ellipsoidea*	Fifty-two percentage increase in total lipid content	Zhang et al., 2014
Malic enzyme (ME)	Putative malic enzyme gene (GenBank accession: XM-002180295.1)	*Phaeodactylum tricornutum*	Lipid content increased by 2.5-folds and reached a record 57.8% of dry cell weight	Xue et al., 2015

Another linked pathway responsible for TAG biosynthesis which occurred in the endoplasmic reticulum. The key enzyme for this biosynthesis pathway is Acyl-CoA: diacylglycerol acyltransferase (DGAT), which is responsible for converting diacylglycerol to TAGs. Russa et al. (2012) used the endogenous genes of *Chlamydomonas reinhardtii* to overexpress DGAT enzyme, but no rise in TAG level was observed. In an another study, the RNAi was altered for the microalga *Chlamydomonas reinhardtii* and clear overexpression of DGAT enzyme was recorded with 34% rise in TAG production (Deng et al., 2012). In 2015, to enhance TAG production, *Chlamydomonas reinhardtii* was genetically modified with DGAT2 from *Brassica napus*. The Nile red staining demonstrated that the new transgenic alga was rich with polyunsaturated fatty acids like α-linolenic acid and the essential omega-3 fatty acids with an increase of 12% in the transformed organism (Ahmad et al., 2015). Multiple desaturases and elongases are another class of enzyme which are responsible for the production of PUFAs from acyl-CoA in the second stage of fatty acid biosynthesis, commonly can be called as TAG production pathway (Gimpel et al., 2015). In one recent study, Δ12-desaturase (NoD12) was overexpressed by the nuclear transformation method for the microalga *Nannochloropsis oceanica* resulting into alternations in fatty acid composition with a substantial increase in arachidonic acid (Kaye et al., 2015). **Table 2B** summarizes these reports concisely.

**Table 2B:** Metabolic engineering approaches for enhanced TAG biosynthesis in various microalgae.

Target enzyme	Gene source/gene modification technique	Receiver species	Findings	Reference
Acyl-CoA: diacylglycerol acyltransferase (DGAT)	Endogenous gene	*Chlamydomonas reinhardtii*	No increase in TAG levels	Russa et al., 2012
Acyl-CoA: DGAT	RNAi alterations	*Chlamydomonas reinhardtii*	Thirty-four percentage increase in TAG production with one gene	Deng et al., 2012
Δ4- desaturase	Nuclear overexpression microRNA alterations	*Chlamydomonas reinhardtii*	Increased the production of 16:4 fatty acid in transformed line	Zäuner et al., 2012
Acyl-Coa: DGAT 2	*Brassica napus*	*Chlamydomonas reinhardtii*	α-linolenic acid and omega-3 fatty acids was increased of 12% in the transformed organism	Ahmad et al., 2015
Δ12-desaturase (NoD12)	Nuclear transformation by electroporation technique	*Nannochloropsis oceanica*	substantial increase in arachidonic acid in TAG	Kaye et al., 2015

Another promising approach to induce the lipid metabolism rate is to block some targeted metabolic pathways that could lead to the synthesis of energy storage biomolecules mainly starch. In a study of Ramazanov and Ramazanov (2006), a novel starchless mutant of *Chlorella pyrenoidosa* was found to accumulate 20.4% more polyunsaturated fatty acids (PUFAs) compared to its parental *C. pyrenoidosa* 82T. Under nitrogen starvation, this starchless mutant showed lipid content of 38% (dcw) only against 25.2% control.

## Mass Cultivation

The idea of microalgae mass cultivation as a source of biomass for various value-added products originated more than one century back in Warburg’s laboratory (1883–1970). Microalgae mass cultivation carries tremendous importance as enormous biomass is required for commercial production of biodiesel from microalgae. Two types of mass cultivation systems are currently available: (i) enclosed photobioreactors, and (ii) open-pond culture system. The former is of more regulated type and successful in maintaining the monoculture of microalgae. Monoculture of *Chlorella*, *Spirulina*, and some other microalgae are successfully cultured in tubular and flat plate photobioreactors.

Open raceways would rather be more preferred for microalgae mass cultivation for biodiesel purpose. However, achieving high productivity and maintaining mono-algal species are real shortcomings of this system. In open raceways, monoculture of algae is usually achieved by maintaining an extreme culture environment, such as high salinity and high alkalinity, as in the case of *Dunaliella* and *Spirulina*, thus indicating that limited microalgal species could be cultivated in such system. The real challenge here is to find out the measures to grow monocultures of lipid-rich strains preferring to grow under standard nutritional/environmental conditions. Moreover, high biomass productivity is the main milestone to be reached for making algae-biodiesel a commercial reality.

In a study, using wastewater containing 85–90% carpet industry eﬄuent and 10–15% municipal sewage, Chinnasamy et al. (2010) had grown a consortium of 15 native algal isolates in 950 L capacity raceway ponds. The biomass yield was recorded as 9.2–17.8 tons ha^-1^ year^-1^ with the lipid content of 6.8% (dcw). In another study, Chiaramonti et al. (2013) cultured the green microalga *Nannochloropsis* sp. in a 10 m long (3000 L culture volume) raceway pond and reported an areal biomass productivity of 14.1 g m^-2^ d^-1^. The lipid yield was also reported as 10 tons ha^-1^ year^-1^, which is significantly a high value in comparison with many previous studies. Further, the chlorophycean microalga *Chlorella vulgaris* was grown in a 14.62 billion liters capacity raceway pond at 30 cm depth. In this study, it was observed that the biomass yield was 0.5 g L^-1^ with an areal productivity of 15 g m^-2^ d^-1^. The lipid content was found to be 25% (dcw) (Rogers et al., 2014). In continuation, Raes et al. (2014) reported that the microalga *Tetraselmis* sp. MUR-233 was grown well in a mass cultivation process of 20 cm culture depth with CO_2_ supplementation and the maximum areal biomass productivity was recorded as 15 mg L^-1^ day^-1^. In this case, the maximum lipid content was also found to be 36.4% (dcw) without CO_2_ supply, and 46.5% (dcw) with CO_2_, respectively. Today, the long-term production rate being 8.77 g (dcw) m^-2^ day^-1^ at Dortmund, Germany; 17 g (dcw) m^-2^ day^-1^ at Musina, South Africa, and 30 g (dcw) m^-2^ day^-1^ at China. A much higher value [200 g (dcw) m^-2^ day^-1^] has been projected by Grobbelaar (2009) with a maximum photosynthetic efficiency of 8%. Therefore, a lot of engineering interventions are still required for improvement in pond design, mixing of cultures, nutrient management, efficient sunlight utilization, etc., to reach this theoretical value.

## Harvesting of Microalgal Biomass

Harvesting of microalgae is another area to be addressed critically. Solid–liquid separation may be achieved by centrifugation, however, with prohibitive cost. The addition of chemical flocculants like FeCl_3_ and AlCl_3_ can accelerate the sedimentation process but at the cost of the environment. In order to minimize the environmental damages, less toxic and cheaper flocculants could be better choices.

Bilanovic and Shelef (1988) used various organic polymers such as chitosan, zetag63, zetag92, and N-100. Chitosan was found to be superior for flocculation of the freshwater microalga *Chlorella* sp. and marine *Isochrysis galbana*. Knuckey et al. (2006) developed a rapid method by pH adjustment of the medium, and the addition of Magnafloc LT-25, a non-ionic polymer, and were successful in harvesting a number of microalgal species. Raising the medium pH also found effective for flocculating marine as well as freshwater microalgae (Wu et al., 2012). Studies conducted with various alkaline salts such as NaOH, KOH, NH_4_OH, Ca(OH)_2_ and Mg(OH)_2_ showed that the flocculation amount is a function of logarithm of cell density, and the most cost effective would be the use of mixed calcium and magnesium hydroxides that are extracted from slaked limestones (Schlesinger et al., 2012).

**FIGURE 1:**
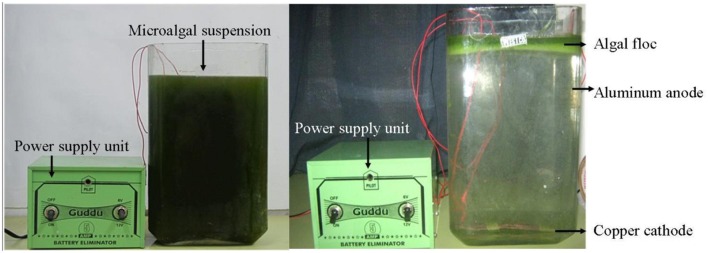
**A laboratory model showing Electro-Coagulation–Flocculation of microalgae**.

In recent years, various new techniques are also emerging in the field of microalgal harvesting. Electro-coagulation–flocculation (ECF) emerged to be an effective method for harvesting microalgal species. In ECF aluminum anode was found to be more efficient than an iron anode. Microalgal suspension rapidly flocculates in higher current densities, but in lower current density, the power consumption and release of aluminum were found to be lower. The harvested biomass had less than 1% aluminum content, but the processed water had aluminum concentration below 2 mg L^-1^ (Vandamme et al., 2011). Although ECF was found to be highly successful in laboratory culture condition, the main challenge is to see its effectiveness under large-scale culture conditions. **Figure 1** presents a clear image of ECF under laboratory condition.

Milledge and Heaven (2013) extensively reviewed various microalgal harvesting techniques, viz., sedimentation, flocculation, filtration, floatation, and centrifugation. Sedimentation of microalgae is dependent on the gravitational forces, which separates out the solid particles from the liquid phase, having different densities. The review also compared various settling rates with respect to different microalgal species. The settling rate can be increased if conjugated with flocculation techniques. The algal cells aggregate due to flocculation and the increased size helped in the better settling of the microalgal flocs. Various flocculants, both organic and inorganic, were studied; however, the use of flocculants was found to be algal specific and the recovery and reuse of the flocculants were found to be problematic. The use of inorganic flocculants was found to be more challenging as the reuse of growth medium was not possible. Flocculants derived from renewable plant and animal sources were also found to be effective in the view of medium reuse but was not viable due to the increase in the flocculants cost. In comparison to flocculation and sedimentation, floatation was found to be effective and relatively fast. Electroflotation and dissolved air floatation was found to be effective and well-known processes for microalgal harvesting. Filtration techniques, viz., membrane filtration, micro-filtration, ultrafiltration, rotary vacuum filters are used for microalgal harvesting but it was found satisfactory for recovering large algal cells, but is not viable due to the reason of low through output and filter clogging. Centrifugation was found to be an effective procedure for microalgal harvesting for all types of microalgae without any hurdles, but the energy consumption was very high with respect to other protocols. This review thus, concludes the non-existence of any harvesting method or combination of harvesting methods suited to all microalgal species, and points significant impact of microalgal harvesting on the design and operation of both upstream and downstream processes.

Bilad et al. (2013) proposed an economically viable method by the use of magnetically induced membrane vibration system (MMVS), which also controls the fouling of biomass. This system consisted of two flat-sheet membranes with different porosity and they were fixed to a module frame to which the vibration frame was connected. An engine was connected to the whole system. The vibration was generated through the engine by a magnetic force which was developed via a pull-and-push mode, by the help of a programmable sonic device. Energy consumption in MMVS is minimum as shear stress was experienced only by the liquid-membrane interface and not by the bulk liquid. This process works efficiently in submerged membrane bioreactors and also in the case of direct sewage. MMVS system consists of a vibration driver, vibration engine, and the electric wire. The vibration generated by the magnetically induced vibration enhanced the shear stress acting on the liquid-membrane interface. The vibration driver provided the vibrating signal which was developed by the magnetic attraction and repulsion forces in the push and pull mode. The membranes were periodically cleaned by the use of tap water and soaking in 1000 ppm sodium hypochlorite (NaClO) and 1000 ppm of citric acid (C_6_H_8_O_7_) solution, each for 1.5 h, in between the sequence of filtration tests. In this system, the energy consumption to harvest microalgae was relatively low corresponding to the harvested biomass.

**FIGURE 2:**
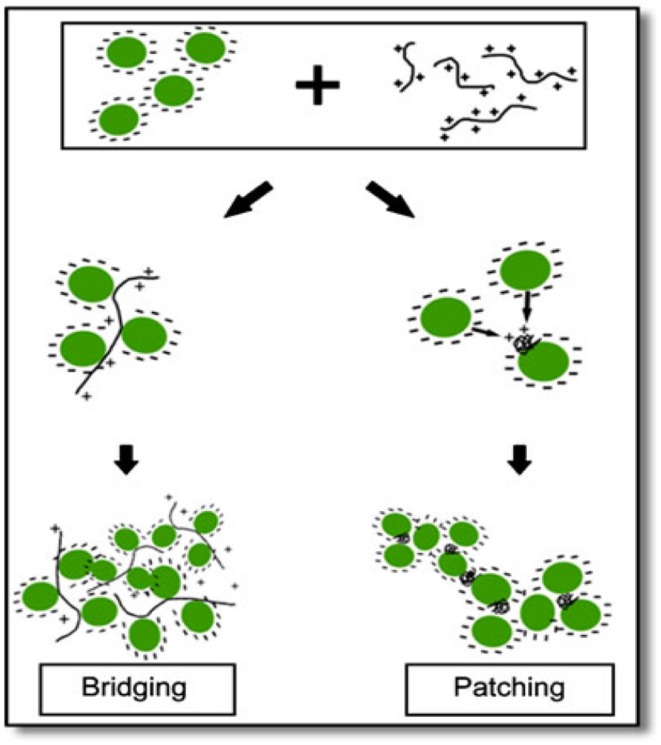
**Schematic view of ‘bridging and patching’ mechanism [reproduced with permission from (Salim et al., 2011)]**. ©Springer (2011).

A novel harvesting technique, bio-flocculation was proposed by Salim et al. (2011) in which flocculating microalgal species was used to harvest microalgal species non-flocculating in nature. This method has enormous advantages: (i) chemical flocculants was not used, therefore, ecofriendly, (ii) Both the microalgal species can be grown in the same growth conditions, thereby not disturbing the growth conditions of microalga of interest, and (iii) no pre-treatment of the medium for reuse. The mechanism proposed for this process is ‘bridging and patching’ by the cationic polymers excreted by the flocculating microalga (**Figure 2**). Microalgal cells bind up with positively charged polymers, either partially or entirely. In partial binding, the microalgal cells bind up with the empty portion of the polymers, thus bridging them and a network is formed of microalgal cells and polymers. The microalgal suspension samples were taken in a cuvette and diluted. The algal suspension was mixed thoroughly and was kept in darkness at 27°C to settle down in a spectrophotometer. pH and temperature were calculated at the starting and also in the end for all samples and were found to be constant, respectively, at 27°C and pH 7.0. The sample turbidity was calculated at 750 nm during the settling period at the same height in the cuvette to determine the microalgal recovery. The results showed that faster sedimentation can be obtained by the addition of auto-flocculating microalgae into the non-flocculating microalgal species, thus increasing the harvesting efficiency. As usual, the technique has not been checked in large-scale ponds and the medium being now contaminated with the non-desired (auto-flocculating) species, whether can be usable for cultivation of the desired one needs to be explored extensively (it has to be noted here that flocculation efficiency of 70% was recorded in this study).

**Table 3** presents an overview of various harvesting techniques with their efficiency used for microalgae harvesting. Although in most of them (flocculation efficiency) seem to be very attractive and efficient under laboratory culture condition, the challenge lies here to maintain their efficiency for bulk harvest without contaminating the growth medium and ensuring minimum environmental damages.

**Table 3:** Reports on various harvesting techniques used for microalgae harvesting.

Organism	Harvesting technique	Flocculation efficiency (%)	Reference
*Chlorella* sp.	Cationic polymer	65–95	Bilanovic and Shelef, 1988
*Chaetoceros calcitrans* *Chlorella muelleri* *Thalassiosira pseudonana* *Attheya septentrionalis* *Nitzschia closterium* *Skeletonema* sp. *Tetraselmis suecica* *Rhodomonas salina*	Non-ionic polymer	80	Knuckey et al., 2006
*Chlorella vulgaris* *Scenedesmus* sp. *Chlorococcum* sp. *Nannochloropsis oculata*, *Phaeodactylum tricornutum*	High pH	90	Wu et al., 2012
*Chlorella vulgaris*	Electro-coagulation	95	Vandamme et al., 2011
*Chlorella vulgaris* *Scenedesmus obliquus* *Neochloris oleoabundans* *Tetraselmis suecica* *Ankistrodesmus falcatus*	Bio-flocculation	70	Salim et al., 2011
*Nannochloris* sp.	Calcium hydroxide	>90	Schlesinger et al., 2012
*Phaeodactylum tricornutum, Chlorella vulgaris*	Magnetically induced membrane vibration (MMV) system	97	Bilad et al., 2013

## Drying

After harvesting, removal of water from the wet biomass is necessary for storage of the algal feedstock and downstream processes. Generally, wet microalgae consist of >80% moisture content. To heat and evaporate the water from a temperature of 20°C at the atmospheric pressure requires an approximate energy input of 2.6 MJ kg^-1^ or over 700 kWh m^-3^ (Milledge and Heaven, 2013). It is estimated that microalgal drying can account up to 30% of the total production costs of algae to fuel. The intensive energy utilized in drying is a big hurdle to commercialize the microalgal biodiesel. Generally, heat is used to dry the wet algal biomass and the sample should not be thick too much for which heat cannot penetrate fully inside the wet samples. Although microalgal drying was considered as a high energy input process, the process is essentially needed for efficient lipid extraction for fuel production.

As the wet microalgal biomass has high moisture content, the drying efficiency is very low in conventional natural drying, i.e., sun drying. Many artificial drying techniques are in use in food industries like drum, spray, freeze, oven, tray, solar, cross-flow, vaccum drying, etc. Freeze drying is a gentle drying process, but it cannot be used in industrial scale as it is a very high energy consuming and slow drying process, whereas spray drying is comparatively fast and efficient but it is a very cost-intensive process. However, there are only a few reports available on microalgal drying and researchers are mainly using oven drying with varying drying time and temperature (**Table 4**).

**Table 4:** Reports on drying methods and temperature used for microalgal drying.

Microalga	Drying method and temperature	Drying time	Reference
*Chlorella protothecoides*	Oven drying at 125°C	24 h	Miao and Wu, 2004
*Chlorella vulgaris*	Oven drying at 60°C	12 h	Widjaja et al., 2009
Fresh water algae	Hot air stream	2 h	Vijayaraghavan and Hemanathan, 2009
*Chlorella* sp.	Oven drying at 105°C	Till to reach constant weight	Haque et al., 2012
*Chlamydomonas reinhardtii* CC-124 wild-type mt(+)	Air drying followed by oven drying at 80°C	48 h	Cakmak et al., 2012
*Chlorella* sp.	Oven dried at 50°C	Till to reach constant weight	Agwa et al., 2012
Freshwater algae	Oven drying at 125°C	12 h	Jernigan et al., 2013
*Nannochloris eucaryotum*	Oven drying at 105°C	24 h	Concas et al., 2013
*Scenedesmus* sp.	Oven dried at 80°C, 5–7.5 mm initial thickness, 10% end moisture content	8 h	Bagchi et al., 2015

In the year of 2004, Miao and Wu (2004), dried the heterotrophically grown microalga, *Chlorella protothecoides* in an oven at 125°C for 24 h and reported optimum oil yield. By contrast, for *Chlorella vulgaris*, drying at low temperature under vacuum gave the best results; the yield decreased significantly when the microalgal samples were subjected to dry at 80°C or above temperatures. Higher temperature can also decrease the total triacylglycerols (TAGs) recovery. At high temperatures, the oxidation of fatty acids takes place, and the unsaturated mainly the poly-unsaturated fatty acid compounds have a much higher susceptibility to oxidation than the saturated fatty-acid groups. As microalgal biodiesel comprises both saturated and unsaturated fatty-acid ester compounds, the TAGs content was affected at a higher temperature of drying (Widjaja et al., 2009). Contrary to this, Haque et al. (2012) dried the cell pellet in an oven at 105°C till to reach the constant weight for lipid extraction from *Chlorella* sp. Jernigan et al. (2013) also studied the biodiesel production using freshwater algae, where the sample was autoclaved at 121°C and 20 psi steam pressure for 1 h followed by heating overnight in an oven at 125°C. The dried mass can be stored in air-tight plastic bags for a long time. Concas et al. (2013) followed oven drying at 105°C for 24 h in case of *Nannochloris eucaryotum*. Conversely, Agwa et al. (2012) and Cakmak et al. (2012) used a low temperature for microalgal drying (**Table 4**).

Vijayaraghavan and Hemanathan (2009) dried the freshwater algae under hot air stream and concluded that the optimum drying period was 2 h, and a further increase in the drying time resulted into lower lipid yield. A report from Utah State University indicated that to reduce the energy consumption and time of drying, the lipid extraction could be done with higher moisture content, i.e., up to 50% moisture remaining in samples. Accordingly, solar drying is the foremost method that has been suggested to be used commercially as it is intrinsically cheap; conversely, drying of hefty algal biomass would require the use of a substantial land area. The report also emphasized that a better technique might be to expand processes that make use of cheap waste-heat generated from the CO_2_ sources like coal or power plant industries (Muhs et al., 2009).

Therefore, there is an obvious necessity to set one detailed investigation report for drying of microalgae in a particular drier which can be used for industrial scale operations, i.e., the exact drying temperature with respect to drying time and a correlation to the final lipid yield. With this concern in mind, one microalgal drying protocol was developed for an indigenous chlorophycean microalga *Scenedesmus* sp., which was dried in a laboratory grade oven at different drying temperatures, viz., 60, 80, and 100°C with varying initial sample thickness such as 5, 7.5, and 10 mm. Lipid recovery was found to be more than 90% when the sample was partially dried with 10% end moisture content. This report also revealed that the power consumption was reduced to half for partially dried samples (Bagchi et al., 2015).

However, for tropical and sub-tropical countries, solar dryers carry immense importance. The basic principle of these dryers is relatively unfussy: heat or warm the air above the ambient temperatures: to about 50–60°C and pass the warm air over the material need to be dried. In most cases, a natural convection principle is used, i.e., the hot air goes upward and low air downward. So, the material has to be kept above a black absorber plate, in an insulated box for drying. The conventional classification of the solar dryers is generally done with the mode of air flow into natural and forced convection dryers. Simple dryers with natural convection mode have not required a fan for the pumping of air from the dryer system. Therefore, research efforts should be focused on designing and constructing a simple natural convection dryer as it does not require any kind of input of energy. The use of solar drier is often been suggested to dry the sample slurry as it is totally pollution free. Thus, the efficient development is needed to construct and model solar dryers for tropical and subtropical countries which is still one of the challenges for the engineers/scientists in the field of microalgal drying.

## Oil Extraction and Transesterification

Like microalgal harvesting and drying, several methods are now in use by different researchers for microalgal lipid extraction, such as cell disruption by bead-beater or sonication, followed by solvent extraction mainly with chloroform–methanol, pre-treatment of biomass with propanol followed by sonication, oil extraction using *n*-hexane in a soxhlet apparatus, lyophilized biomass followed by two-stage solvent extraction, etc. Various cell disruption techniques (autoclaving, bead-beating, microwave treatment, sonication, and NaCl treatment) were compared by Lee et al. (2010); the microwave pre-treatment emerged as the most efficient for lipid extraction from microalgae. By contrast, a report showing pre-treatment of algal biomass did not register any significant rise in lipid recovery is also available (Mandal et al., 2013). This shows that a specific lipid extraction protocol for microalgae is yet to be developed.

A comprehensive review on various lipid extraction methods is provided by Kumar et al. (2015), where they discussed all kinds of lipid extraction processes, such as the solvent extraction processes, mechanical processes and the solvent-free extraction processes. These authors commented on the high costs of solvents as the primary constraint of lipid extraction from microalgae. In case of solvent extraction, two methods are mainly in use by various researchers, e.g., Folch method, and Bligh and Dyer method. The most recent and rigorous method was suggested by Matyash et al. (2008), which provides better recovery of almost all major classes of lipids. Methyl-tert-butyl ether (MTBE) was used as a solvent and this method provides the most accurate lipidome profile.

Extraction of lipids was also carried out under various mechanical approaches, viz., expeller press, bead beating, ultrasonic-assisted extraction, electroporation, and application of microwave radiation. Use of mechanical methods, though environment friendly and cheap, are not a wise option because of poor lipid recovery. Currently, the solvent-free extraction techniques are gaining importance in the field microalgal lipid recovery as innovative and alternative methods. Use of osmotic pressure, isotonic extraction with ionic liquids (called as green solvents), and enzyme-assisted extraction such as use of cellulase and trypsin for disruption of cell wall appear to be promising and ecofriendly alternatives to the organic solvents. These solvent-free methods, though demonstrated high potential at the laboratory scale, further research is required for successful implementation of these technologies at the production scale.

Transesterification has now been widely used to reduce the high viscosity of oils. Catalysts, particularly very strong acids or bases or combination of both, and even lipase enzyme was used to trigger the transesterification process. In recent years, more focus is given to *in situ*/direct transesterification, where the lipid extraction step could be eliminated. Supercritical methanolic conditions such as transesterification at high temperature and pressure with a very high concentration of methanol were used by Patil et al. (2011) with wet algal biomass. *In situ* transesterification studies conducted by Velasquez-Orta et al. (2012) and few others also demonstrated very high concentrations of methanol requirement. Thus developing an environment-friendly and economically feasible *in situ* transesterification process for microalgal biodiesel conversion is the ultimate challenge of the present day.

## Future Perspectives

Microalgal biodiesel can reach its heights with a lot of interventions and advancements at each and every step. One of them could be the metabolic engineering with recombinant DNA technology toward genetically modifications or alterations of the existing lipid/triglyceride biosynthetic pathways resulting into oil-rich microalgal strains. Development of various large-scale photobioreactors designed for commercial scale algae cultivation by utilizing CO_2_ from flue gas emitted directly from industries could also be looked into. Hydrothermal liquefaction could also be explored thoroughly to produce crude oil directly from the wet algal biomass, thereby eliminating the drying, oil extraction and transesterification steps *vis-a-vis* removing the solvent requirements.

Cost is the key roadblock for algae-based fuel as this third generation biofuel feedstock requires a high initial investment for its production. However, combating the challenges in each and every step would certainly reduce the cost of biodiesel production from microalgae. Nonetheless, one of the strategies for cost reduction is producing microalgal biodiesel by using a biorefinery based production strategy, where each and every component of the biomass can be used to produce valuable products. Microalgae are rich sources of various chemicals. Polyunsaturated fatty acids (gamma-linolenic, arachidonic, eicosapentaenoic, docosahexaenoic, etc), pigments (astaxanthin, beta-carotene, etc.), hydrocarbons and various pharmaceuticals with antibacterial, antimycotic, antitumoral, and immunomodulatory properties are found to be accumulated in substantial amount in many species. Integrating the recovery of these high-value products with microalgal biodiesel would certainly bring the algal biodiesel technology to commercial reality. Furthermore, combining microalgae cultivation with wastewater treatments and CO_2_ sequestration from thermal power stations are important milestones to be reached for a clean and sustainable future.

## Author Contributions

All authors listed, have made substantial, direct and intellectual contribution to the work, and approved it for publication.

## Conflict of Interest Statement

The authors declare that the research was conducted in the absence of any commercial or financial relationships that could be construed as a potential conflict of interest.
